# Health Monitoring of Air Compressors Using Reconstruction-Based Deep Learning for Anomaly Detection with Increased Transparency †

**DOI:** 10.3390/e23010083

**Published:** 2021-01-08

**Authors:** Magnus Gribbestad, Muhammad Umair Hassan, Ibrahim A. Hameed, Kelvin Sundli

**Affiliations:** 1 Department of ICT and Natural Sciences, Norwegian University of Science and Technology (NTNU), Larsgårdsvegen 2, 6009 Ålesund, Norway; muhammad.u.hassan@ntnu.no; 2 Cognite AS, Oksenøyveien 10, 1366 Lysaker, Norway; kelvin.sundli@gmail.com

**Keywords:** anomaly detection, prognostics and health management (PHM), predictive maintenance, explainable deep learning

## Abstract

Anomaly detection refers to detecting data points, events, or behaviour that do not comply with expected or normal behaviour. For example, a typical problem related to anomaly detection on an industrial level is having little labelled data and a few run-to-failure examples, making it challenging to develop reliable and accurate prognostics and health management systems for fault detection and identification. Certain machine learning approaches for anomaly detection require normal data to train, which reduces the need for historical data with fault labels, where the main task is to differentiate between normal and anomalous behaviour. Several reconstruction-based deep learning approaches are explored in this work and compared towards detecting anomalies in air compressors. Anomalies in such systems are not point-anomalies, but instead, an increasing deviation from the normal condition as the system components start to degrade. In this paper, a descriptive range of the deviation based on the reconstruction-based techniques is proposed. Most anomaly detection approaches are considered black box models, predicting whether an event should be considered an anomaly or not. This paper proposes a method for increasing the transparency and explainability of reconstruction-based anomaly detection to indicate which parts of a system contribute to the deviation from expected behaviour. The results show that the proposed methods detect abnormal behaviour in air compressors accurately and reliably and indicate why it deviates. The proposed approach is capable of detecting faults without the need for historical examples of similar faults. The proposed method for explainable anomaly detection is crucial to any prognostics and health management (PHM) system due to its purpose of detecting deviations and identifying causes.

## 1. Introduction

**Figure 1:**
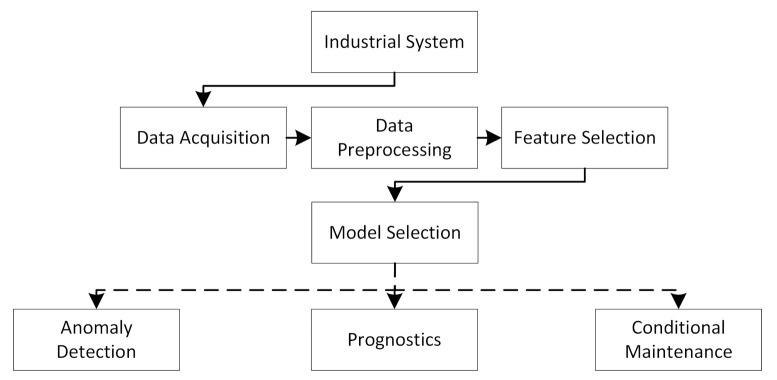
An illustration of prognostics and health management system using deep learning models.

Prognostics and health management (PHM) has become a popular topic. It concerns finding the actual condition of a system and preferably predicting when it will fail. According to Goebel [1], a successful PHM system should be able to answer the following questions: (1) Is there something wrong with the system? (2) If so, what is wrong? and (3) When will it fail? Having such a system is becoming increasingly important in many sectors. The major tasks of a PHM are listed in Figure 1. For instance, in order to successfully achieve fully autonomous ships, a system that can answer these questions about vital equipment is crucial. Typically, in order to answer question two and three, a lot of labelled data and run-to-failure examples is necessary. Collecting these kinds of data is often challenging, and building such a system can therefore be demanding. The first question can, on the other hand, be explored with less labels and fewer examples of failure. Due to its less data-hungry nature, this work mainly focuses on answering the first question for maritime starting air compressors. Starting air compressors are used to start a ship’s main engines and thus are vital to a ship’s operation and the safety of personnel, material, and in the worst case, the environment. With an air compressor’s criticality, they are conservatively and rigorously maintained, leading to extremely few examples of real-world failures. However, if one could, based on the data from normal operation, detect abnormal behaviour, two concrete condition-based actions can be taken: (1) The compressor can be maintained before it fails and (2) the conservative maintenance intervals can be increased, in order to save costs.

The second focus area of this work is how to get more explainable results. Explainable results from anomaly detection algorithms have two important advantages: (1) It helps the service personnel locate the misbehaving parts of the system and (2) it helps in increasing the system operator’s trust in the predictions.

Anomaly detection has been used in various applications, such as fraud detection, cybersecurity, condition systems, and much more. Abnormalities, novelties, and outliers are other terms used interchangeably with anomalies. Anomalies are typically divided into the point, contextual/conditional, and collective or group anomalies [2]. In prognostics and health management (PHM), anomalies often refer to the deviation of expected behaviour, which is used to indicate the monitored system’s condition [3]. In this project, anomaly detection is used to detect abnormal behaviour, indicating an air compressor’s condition. Classification and anomaly detection are closely related, but the classification is typically used for supervised training [2].

The rest of this work presents a review of several state-of-the-art papers in Section 2. However, Section 3 provides a thorough understanding of deep learning-based models used in PHM. Section 4 delivers information about data collection for this work. The methodology of this work is available in Section 5. Section 6, Section 7 and Section 8 are about model configuration, results, and model transparency, sequentially. Finally, Section 9 and Section 10 discusses and concludes this work, respectively.

## 2. Related Work

In general, anomaly detection is closely related to diagnostics, but in PHM it is treated as two different approaches. Often anomaly detection differs from traditional fault identification based on whether labels are available or not. Multiple deep learning (DL) techniques have been explored for anomaly detection. Park et al. [4] used a long short-term memory (LSTM)-based variational autoencoder (VAE) for detecting anomalies in a robot-assisted feeding system. The approach received a higher accuracy than other methods such as one-class support vector machine (OSVM) and autoencoder (AE). In 2015, Malhotra et al. [5] proposed a method for detecting anomalies in a time-series based on stacked LSTM networks. The network is trained on normal data exclusively, and predictionEntropy 2021, 1, 0 3 of 27 errors are evaluated based on Gaussian distribution to find the probability of abnormal behaviour. Malhotra et al. [6] used a reconstruction-based anomaly detection approach with LSTM to detect anomalies in multi-sensor data. The model was trained only with normal data, and the results indicated that it was successfully able to detect anomalies in several datasets.

Several AE-based algorithms have been applied successfully for anomaly detection. Yan and Yu [7] proposed a method based on stacked denoising autoencoder (DAE) combined with a supervised classifier to detect anomalies in a gas turbine combustor. Another approach used VAE to do anomaly detection based on reconstructed probabilities [8]. They used these probabilities to classify if samples were anomalies or not. Their results showed that the VAE approach performed better than AE-based and principle component analysis (PCA)-based methods. Anomaly detection with AEs or other encoder-decoder (ED) architectures is typically done by training on normal data, leading to good reconstructions of normal large reconstruction errors on abnormal data. In 2019, Ellefsen et al. [3] proposed an unsupervised reconstruction-based algorithm for detecting faults in maritime components. Their approach is based on machine learning (ML) algorithms with ED-architecture. The results proved that the maximum acceleration in the reconstruction error could detect faults without having labels.

Deep belief network (DBN) has also been used for anomaly detection. Wulsin et al. [9] proposed the use of DBN to measure an anomaly score on clinical electroencephalography (EEG) images from humans. Their approach showed that a DBN can be used effectively to measure anomalies. Zenati et al. [10] proved that generative adversarial network (GAN) can also be used effectively to detect anomalies in high-dimensional data. They tested the model on a dataset with handwritten digits and one with network intrusions. Li et al. [11] also used GAN for anomaly detection, but on multivariate time series data. In order to capture potential anomalies across time-steps, the proposed method used LSTM as a GAN. Lim et al. [12] highlighted the problem of having few examples of anomalies in a dataset and proposed a method based on GAN called an adversarial autoencoder (AAE) for data augmentation. The idea is that the model will create low-dimensional latent distribution from where samples can be drawn. Instead of drawing samples based on the probability, which will create a lot of normal data, the samples are systematically drawn from the probability distribution hence, generating anomalies.

Much work related to detecting anomalies in systems and data is presented. The literature indicates that reconstruction-based approaches are promising in detecting abnormal behaviour. This project compares and explores the use of LSTM, convolutional neural network (CNN), DBN, and variants of AEs to detect anomalous behaviour in air compressors. The next chapter contains a brief theory of the relevant approaches.

## 3. Theory-Deep Learning Models

**Figure 2:**
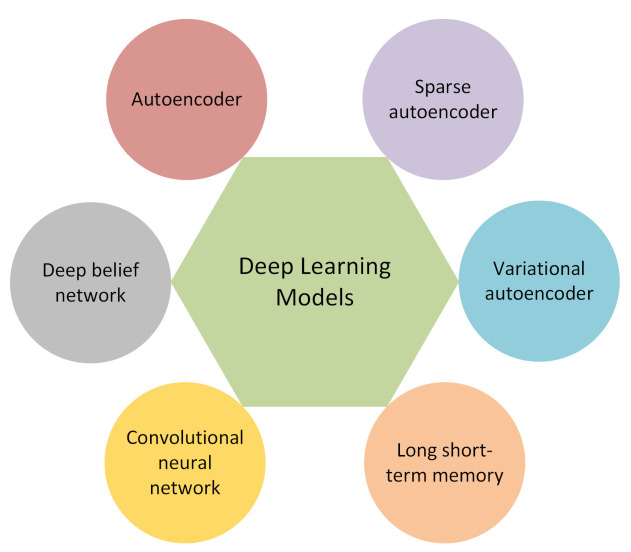
Six different types of deep learning models applied for anomaly detection.

Several different types of deep learning approaches have been applied to problems related to anomaly detection. In this section, the approaches applied in this project are introduced. Figure 2 provides an illustration on six deep learning models that were used to investigate the anomaly detection in the context of the prognostics and health management of air compressors.

### 3.1. Autoencoders

An AE is an unsupervised approach based on artificial neural networks (ANN) [13]. In general, an AE is a feed-forward neural network (FNN) with an input layer, output layer, and one or more hidden layers. It is trained to copy its inputs to its outputs through the hidden layer(s). Hidden layers are considered a bottleneck that forces the network to make a dimensionality reduction of the inputs and thus finds which characteristics are important in the data [14]. Compared to traditional methods such as PCA, AE can learn non-linear transformations.

AEs have been applied to solve several types of problems, often around feature extraction, noise reduction, dimensionality reduction, and anomaly detection. Speech enhancement (removing noise) [15], natural language processing [16], and images [17] are examples of problems where AE has been applied. It has also been applied in PHM for feature extraction [18] or in combination with a supervised layer to do fault diagnostics [14].

### 3.2. Sparse Autoencoder

Sparse autoencoder is a variant of the AE which uses an alternative approach that does not require a reduced number of neurons in the hidden layer(s) to provide the bottleneck. Instead, it uses a loss function that penalises activations in the hidden layer(s) [19]. The idea is that the network learns encoding and decoding, which relies on a small set of total neurons, limiting the capacity of memorisation.

The basis of the VAE is that the encoder outputs a probability distribution for each extracted characteristic of the input data, instead of giving each of the characteristics a value [19]. The distribution is assumed to be normally distributed, which means a probability distribution can be represented only by the mean and standard deviation. After the encoding, the input data is represented only as a set of means and standard deviations. A random sample from each of these probability distributions is passed to the decoder model. The decoder model will then try to reconstruct the original input based on these samples.

### 3.3. Long Short-Term Memory

LSTM is a variant of recurrent neural network (RNN) designed to learn long-term dependencies [20]. It introduces the idea of a memory cell, which contains gates that tries to regulate the information through the cell. The result is a network that achieves contextual weights that can deal with long-term dependencies flexibly. Several variants of the LSTM have been introduced, such as the Vanilla LSTM [21] and GRU-LSTM [22]. The Vanilla LSTM has proved itself useful for PHM therefore, it is the preferred variant of LSTM in this project. It has several variants [23]. In this work, the variant without peephole connections was used. The Vanilla LSTM (referred to as just LSTM from now on) has four interacting neural network layers.

The principal of AEs is used in LSTM architecture to build what is referred to as a ED-LSTM. The architecture has a bottleneck to reduce the dimensionality, but compared to simple AEs, the LSTM also considers time.

### 3.4. Convolutional Neural Network

CNNs are a type of DL technique known for their performance on images. They have been used to classify images, cluster images, identify faces, and much more [24]. However, they are often used for images. Nevertheless, they can also be used for 1D data such as a time-series or 3D data such as videos. A CNN is an ANN model that uses convolution operations in at least one layer. CNN has become popular due to its ability to extract important features from input data automatically. One of the motivations for using CNN is that it reduces computation requirements due to weight sharing [25]. A typical CNN consists of four types of layers: Convolutional, pooling, flattening, and fully connected [13].

A CNN can be used in time series sensor data by transforming the data into a 2-dimensional tensor format by concatenating the individual signals from a sliding time window [26,27]. In this project, the CNN is used with ED architecture to enable it for reconstruction-based anomaly detection.

### 3.5. Deep Belief Network

DBN is an unsupervised DL model proposed by Hinton et al. [28] as an alternative to back-propagation. It is considered a generative graphical model composed of multiple layers of hidden units. The layers are connected, but not the units within each of the layers. The architecture of a DBN looks similar to a multi-layer perceptron (MLP), but the building blocks and training process is different. A DBN is a set of stacked restricted Boltzmann machine (RBM)s. It can be combined with a final layer to do classification or regression, or similarly to the AE, it can be used for feature extraction. A DBN can be trained to reconstruct its inputs in a probabilistic manner. For example, it can be used as a features detector before a regression or classification layer [29,30].

## 4. Data

This project is a collaboration with a company providing maritime equipment. Some information, such as time scale, sensors, and fault types is anonymised due to a non-disclosure agreement with the collaborating company.

In the project, 25 datasets consisting of various sensor data from air compressors, are available. Five types of sequences are logged: Fault types A, B, C, D, and sequences where the compressor runs normally. The datasets with fault A, B, and C starts with different operational loads and settings and with the air compressor in a normal condition. At a random point in time in each set, the system starts to degrade. The system degrades until it is out of operating condition and fails. For these three types of faults, the time of which the fault is approximately 33%, 67%, and 100% severity is logged by a domain expert. The system does not fail when a fault has reached 100% severity, but the fault will, with time, degrade the system until failure. Fault D is not related to any complete system failure but a fault related to internal control system communication problems. It was only used to evaluate the proposed anomaly detection approach. The datasets without faults are sequences where the compressor is running in normal operating conditions. Each dataset consists of measurements from 14 sensors and the length of the sequences differs. The sensors are a combination of pressure, temperature, current, and oil sensors to measure important signals in the compressor process. More details about the sensors and faults cannot be disclosed due to the non-disclosure agreement. An overview of the types of sequences is presented in the list below.

- **Fault A:** Data where the compressor starts in a normal condition, but a fault is eventually forced. The approximate time of 33%, 67%, and 100% fault progression is logged. After the 100% fault is introduced, the compressor is running until reaching the criteria for end-of-life;
- **Fault B:** Similar procedure as for fault A, but with another type of fault;
- **Fault C:** Similar procedure as for fault A and fault B, but with another fault condition;
- **Fault D:** Does not start in a normal condition, the fault is present in the entire sequence and is mentioned as related to communication problems. The fault does not force the compressor towards failure;
- **Normal:** Data with no faults that are collected during regular operation.

Table 1 describes the available data regarding how many sets of each type and the length of the sequences. Due to the non-disclosure agreement, the length of the sequences are only given in the number of samples.

For fault A, B, and C, the end of sequence determines the compressor’s end-of-life, which means that abnormal behaviour should be detected before end-of-life, preferably in good time. Even though faults A, B, and C are started in so-called normal conditions, the initial part of the sequence often shows signs of the previous fault. This is due to collecting several sequences with a short time interval. Hence, the compressor has not been able to regain its normal condition before running for a little while.

### Evaluation Data

A randomly-selected and labelled dataset is used for testing the classification performance of the models. The evaluation dataset contains 150 normal samples and 200 anomalous samples from unseen sequences. The presented model gets an accepted prediction if it can correctly identify the input. Hence, the final performance measures the accuracy of the evaluation set. The 150 normal samples are selected from three sets with fault A (50 samples), four sets of fault B (50 samples), and one set with fault C (50 samples). The anomalies are selected from three sets with fault A (50 samples), four sets of fault B (50 samples), one set of fault C (50 samples), and two sets of fault D (50 samples). The normal samples are randomly selected from parts of the fault sequences before any fault is introduced, while the anomaly samples are taken from parts where the fault has reached 100% severity. A random seed is introduced in order to make the evaluation dataset equal for all the models.

## 5. Methodology

The project investigates and compares different types of DL models based on ED architecture to see their ability to detect anomalous behaviour in air compressors. Models that use ED architecture for anomaly detection follow a similar principle. Data in original dimension are passed through a bottleneck, forcing the data into a lower-dimensional representation. The original input is reconstructed based on the compressed representation. The bottleneck forces the model to choose important features that best represents the data. In other words, the models try to reconstruct the input data (both shapes and values). When such models are used for anomaly detection, they are only trained with normal data, resulting in the model providing good reconstructions on normal data but worse reconstructions on anomalous data. The results refer to a reconstruction error, which can essentially be any measure of the error. In this project, the absolute value of the difference between the input, *x*, and the reconstructed input, $\hat{x}$, was used (Equation (1)):

(1) $$\mathrm{Reconstruction}\;\mathrm{error}=\frac{1}{N}\sum_{i=1}^{N}\left|x_{i}-\hat{x_{i}}\right|$$

The idea is that the obtained reconstruction error will be low when the models are presented with normal data, similar to what it has seen before, but higher when the compressor’s health is degrading. The reconstruction error is in an undescriptive range, giving little information on its own.

Four datasets with fault A and three datasets with fault B are used to investigate and define a method for converting the non-descriptive reconstruction error into an informative scale between 0 and 100. The closer to 0, the more normal the data is. It was investigated if a threshold should be obtained to decide if data are normal, anomalous, or in-between. The expected output is an anomaly score that can be used to determine the condition of the system. The proposed method for converting the reconstruction error into a descriptive range is simple and consist of three steps:

(2) $$\sigma \left(z\right)=\frac{1}{1+e^{-z}}$$

1.  Scale the reconstruction error into a range suitable for a sigmoid transformation. In the sigmoid function, a −8 gives approximately 0, and 8 gives approximately 1. The step requires one to decide the minimum and maximum values of both the old and new range;
2.  This step is a sigmoid transformation with Equation (2). In this step, the exponent of the *e* is multiplied with a number between 0 and 1. This value needs to be adjusted to achieve the desired behaviour. It decides the steepness of the transformation;
3.  The final step is to transform the values into a scale between 0 and 100. This step can be skipped if desired since the sigmoid function returns values between 0 and 1.

This transformation was combined with thresholds dividing the range into three classification zones: Normal from 0 to 40, warning from 40 to 60, and danger from 60 to 100. These are configurable but added in order to verify quality and provide describable outputs into potential control systems. These thresholds or classification zones could be avoided by adding a threshold directly on the reconstruction error.

### Evaluation

The proposed approach is not a classifier or a basic regression model, meaning that typical scoring methods such as accuracy or mean square error are not sufficient to describe the performance of the approach. The proposed thresholds allow an accuracy measure to test if the models are able to distinguish between normal and failing equipment. It is, on the other hand, not an adequate measure of the proposed approach. Therefore, visual inspection is necessary and the most important evaluation is to compare the models and evaluate if the proposed method has the desired behaviour.

## 6. Model Configuration

The six DL models explored in this case study need to be designed to fit this particular problem. The models have several adjustable parameters such as the architecture (number of layers and nodes per layer) and other hyper-parameters like an optimiser, activation functions, and learning rate. LSTM and CNN also require one to select a time window to generate the input data format. These parameters were adjusted manually for each algorithm. The process of manual tuning was devoted to finding promising settings for the adjustable parameters. The selection of architecture and parameters was based on finding a model that accurately reconstructs normal data but obtains a gradually increasing reconstruction error as faults are progressing. This process is more challenging to automate than for other typical machine learning approaches since a too-complex (large) model will be able to copy the input to the output. If the model has the right complexity, the model will be able to reconstruct the normal data (training data), but as other data is presented, the model will struggle to reconstruct it.

A tanh activation function was selected for the output, which means the model’s output is between −1 and 1. Therefore the data was normalised using min-max-scaling between −1 and 1. This forces the model to provide inputs on the right scale. The data normalisation was based on the training and configuration sets, and the same transformation was used for the test set. Next, the final architectures and parameters found for each algorithm are presented.

### 6.1. Architecture and Parameters

#### 6.1.1. Autoencoder

**Figure 3:**
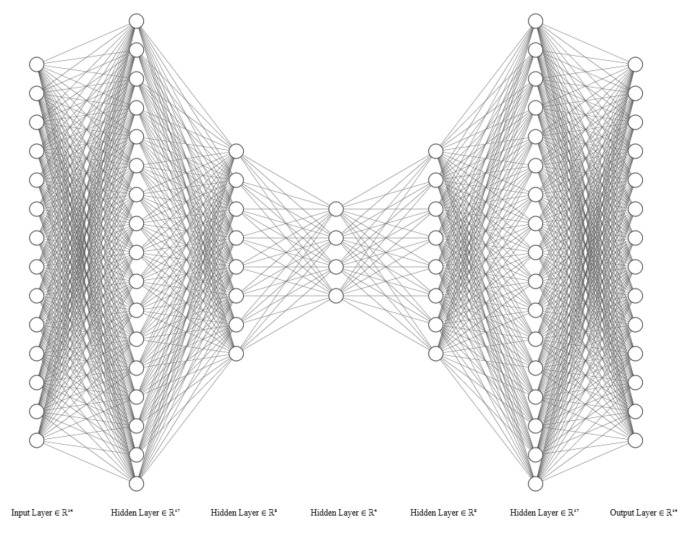
Architecture of the autoencoder (AE) model.

The architecture and parameters for the AE model were found through trial and error. Experiments with four types of optimisers, stochastic gradient descent (SGD), RMSProp, Adam, and AdaGrad, indicated that the performance was quite similar, but RMSProp was slightly better. A learning rate that gave the desired learning curve was chosen to be 0.0001. The default weight initialisation method called Xavier weight initialisation was used. Since the problem is hard to optimise with hyperparameter tuning, several architectures were explored, and the final architectures found are described in Table 2. Figure 3 shows the architecture summarised in the table. The architecture in the other models look similar but adjusted based on the architecture information in each table. The main finding in the architectural choice was that the results were improved when the first hidden layer had increased nodes compared to the input.

#### 6.1.2. Sparse Autoencoder

The sparse autoencoder model’s architecture and parameters have equally many layers as the AE, but every layer has 14 nodes. This is due to the sparsity regularisation, which penalises model complexity. It will lead to a similar effect as a normal AE, but the sparse autoencoder (SAE) learns which and how many nodes to remove based on forcing weights to zero. The same optimiser, learning rate, and weight initialisation approach, as for the AE, was used. The parameters are presented in Table 3.

#### 6.1.3. Variational Autoencoder

The architecture and parameters found for the VAE model is presented in Table 4. The table shows that the latent space (middle layer) contains information from six dimensions. It also has more nodes in the first hidden layer than in the input layer.

#### 6.1.4. Deep Belief Network

The best performing architecture for DBN consists of three stacked RBMs. The input layer has 14 inputs, while the next layers have 16, 13, and 11 nodes, as presented in Table 5. The *k* value used was 10, and a learning rate of 0.003. The predictions from the DBN were smoothed using a moving average filter with a window size of 50.

#### 6.1.5. ED-LSTM

Similar to the other models, the best performing parameters were selected based on trial and error. The LSTM does not only reconstruct the sensors for one time-step, but for all time-steps in the selected time window. This means that the input is a 2D input format containing all the sensor values for each timestep in the selected time window. The goal of the modal is to reconstruct that same input data, in the same format. Table 6 shows selected parameters for the LSTM. The table shows that the SGD with a learning rate of 0.001 performed the best with three hidden layers of 10, 7, and 10. The time window was selected to be 20.

#### 6.1.6. ED-CNN

**Figure 4:**
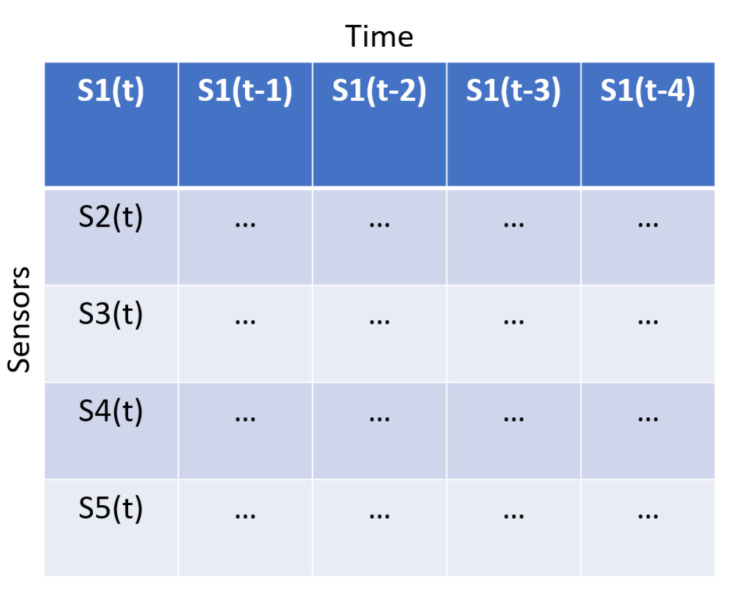
2D input data format to convolutional neural network (CNN) model with time series data.

A CNN has many parameters to tune. A time-window of size 20 gave the most promising results. The time window was used to transform the sensor data into a 2D data object where the y-axis is the sensor and the x-axis is the time. This means that the first row of the data structures contains data from one sensor at each sample within a time window. This is illustrated in Figure 4. Therefore, the input data is formatted as an image, and the model aims to reconstruct the image. The architecture found consists of combinations of convolutional layers (Conv2D) and max-pooling layers for encoding and convolutional layers and upsampling for decoding. Table 7 lists each layer used for the CNN model and its settings. The padding approach called same was used for all convolutional layers. This means that the output is padded to reach the same dimension as the input. The middle layers use the ReLU activation function, but the final layer uses tanh to get the data into the correct scale. The Adam optimiser, with a default learning rate of 0.001 performed the best.

### 6.2. Reconstruction Error

**Figure 5:**
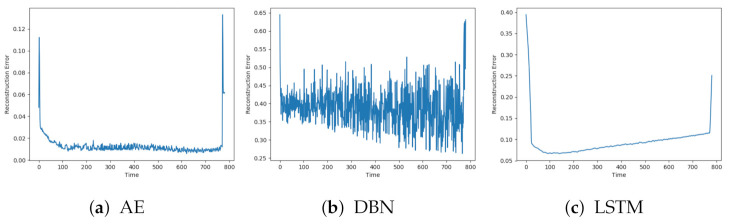
Reconstruction error on sequence with normal data with AE, deep belief network (DBN) and LTSM.

Each model was trained on the available normal sequences. Figure 5a–c show the reconstruction error obtained on one of the normal sequences with AE, DBN, and LSTM, respectively. Each of the reconstructions has a peak at the beginning and end of the sequence. This can be explained by beginning the logging before the compressor starts and terminating after the compressor stops. The models achieve quite different results, both for when it comes to how well the signals are reconstructed and the level of noise. DBN produces a lot of noise and a high reconstruction error compared to the other two models. The AE has a quite low reconstruction error, below 0.02, and some noise. The LSTM model produces even less noise, but with a slightly higher reconstruction error than the AE.

**Figure 6:**
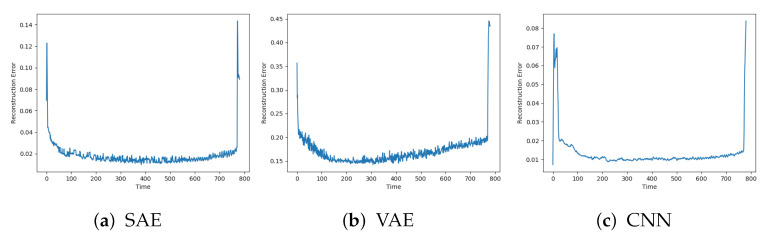
Reconstruction error on sequence with normal data with SAE, VAE and CNN.

The reconstruction error on normal data from the three other models is shown in Figure 6a–c. The results show similar trends as the previous models. The CNN has less noise than the two others. The SAE and CNN have similar level of reconstruction error to the AE, while the VAE has higher.

**Figure 7:**
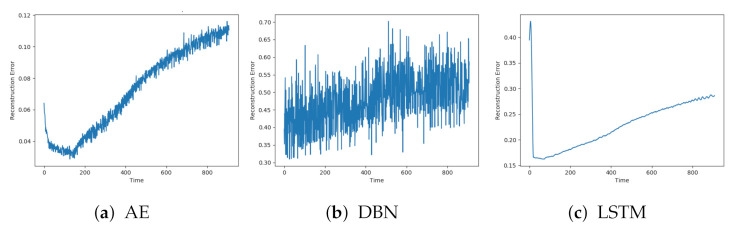
Reconstruction error from AE, DBN, and LTSM on sequence with failure due to fault type A.

Next, the reconstruction errors from the models are evaluated on data with faults. These data sequences start from the normal condition but are gradually introduced for faults until end-of-life. Figure 7a–c shows the reconstructions from AE, DBN, and LSTM on a sequence with fault A. The individual models were affected by similar patterns of noise. The AE and LSTM both start with a low reconstruction error, which increases towards the end of the sequence. It is hard to see any clear trend from the DBN due to the high noise level.

**Figure 8:**
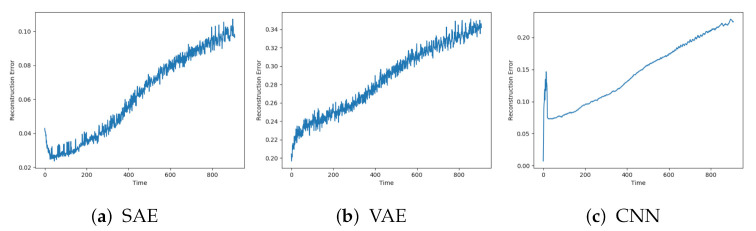
Reconstruction error from SAE, VAE, and CNN on sequence with failure due to fault type A.

Figure 8a–c show the reconstructions from the SAE, VAE, and CNN models on the same data sequence. These models were able to start with a low reconstruction error, then gradually increase until failure.

**Figure 9:**
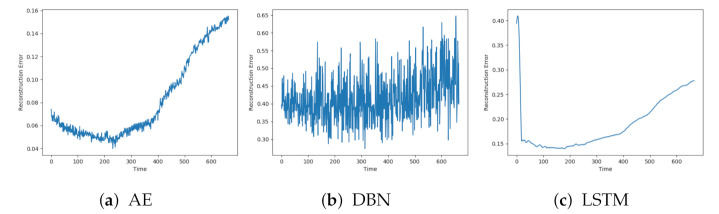
Reconstruction error from AE, DBN, and LTSM on sequence with failure due to fault type B.

Figure 9 indicated that the AE, DBN, and LSTM achieved similar results as seen so far. The AE and LSTM went from a low reconstruction error to an increasing one. It was hard to see a clear trend in the results from the DBN due to the noise.

**Figure 10:**
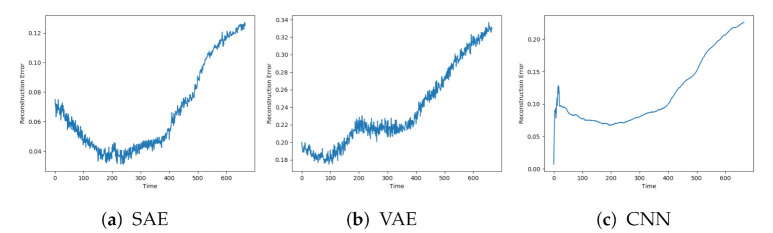
Reconstruction error from SAE, VAE, and CNN on sequence with failure due to fault type B.

The SAE, VAE, and CNN models achieved the desired trend in the reconstruction error. This is illustrated in Figure 10. The beginning of the sequence seems to lead to an increased reconstruction error before going down to the normal level. This can be due to the described data collection process where new data sequences were collected before the compressor had regained the normal condition.

**Figure 11:**
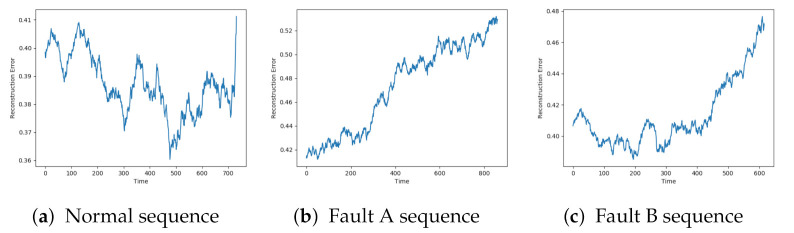
Reconstruction error with a moving average filter obtained from DBN.

All the tested models, except DBN, produced results according to the desired behaviour. A moving average filter is applied to see if the reconstruction error from the DBN model also has an increasing trend. Figure 11a–c show the reconstruction error on the three sequences seen so far, but with a moving average filter with a time window of 50 units. This reveals that the sequences with faults obtain the desired trend from DBN as well.

The results show that all models were able to reconstruct the normal data better than fault sequences, resulting in an increasing reconstruction error close to failure. Each model reproduces the input with a different error rate, ranging from the AE with the lowest and DBN with the highest. In theory, it does not matter if the reconstruction error is high for normal data, as long as it is even higher for failure data. It could be possible to use the reconstruction error directly to indicate if the system is behaving unexpectedly. The scale is not informative, and the only indication of abnormal behaviour is an increasing score. The next section explores if a more descriptive range of the reconstruction error can be obtained.

### 6.3. Transformation

The reconstruction error can be transformed into a more descriptive range between 0 and 100, referred to as an anomaly score. An anomaly score of 100 indicates faulty behaviour, while 0 indicates a normal condition. The range is divided into three zones, which represent the state of the system. These are normal, warning, and the danger zone. The next section describes how transformation and thresholds are decided.

The transformation is tuned individually for each model based on the configuration settings. The tune-able parameters are related to the min-max-scaling and sigmoid transformation, as explained previously. There are three phases of the transformation: (1) To scale the reconstruction error between two values, (2) transform with the Sigmoid function, and finally (3) which is to scale it to between 0 and 100. In step 1, both the old and new minimum and maximum values must be selected. In step 2, the sigmoid exponent must be selected. These values are manually tuned until the desired trend ranging from 0 to 100 is obtained. The outcome of this transformation is referred to as the anomaly score. The parameters that gave the best fit on the configuration set is shown in Table 8.

The goal of the configuration was to get a common scale where the anomaly score indicates the condition of the system. The scale can also be divided into zones. Ideally, one normal zone and one danger zone, with a neutral or warning zone in between. Based on the results from the configuration set, the thresholds are decided to be 0.4 and 0.6. This means that when the anomaly score is below 40, it is considered a normal operating condition while increasing over 60 indicates that something is wrong. These limits are manually selected and need to be adjusted according to the use-case.

**Figure 12:**
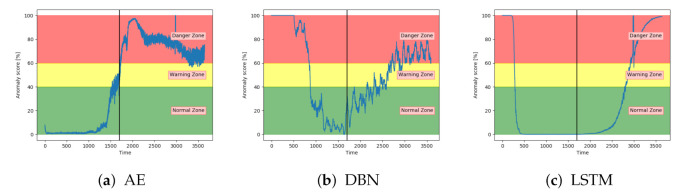
The anomaly score from AE, DBN, and LTSM on sequence from a configuration set with failure due to fault type A.

Figure 12a–c shows the achieved anomaly score from the AE, DBN, and LSTM model, on one of the sequences with fault type A in the configuration set. The normal zone is represented by green, the warning zone by yellow, and the red is the danger zone. The fault is introduced over three levels of severity. The black vertical line in the figures indicates when the fault has 100% severity. The idea with anomaly detection is not about predicting that something is about to fail but to give insight into the current condition. In particular, fault A is according to the available sensor measurements resulting in long-term changes, not sudden impact. The AE starts with an anomaly score close to zero, before gradually increasing towards a peak, then it decreases slightly. The DBN starts with a high anomaly score. It decreases down to the normal zone before increasing towards the danger zone, reaching it just before it fails. The LSTM model starts similar to the DBN but quickly finds the normal level before gradually increasing after the 100% error is introduced.

**Figure 13:**
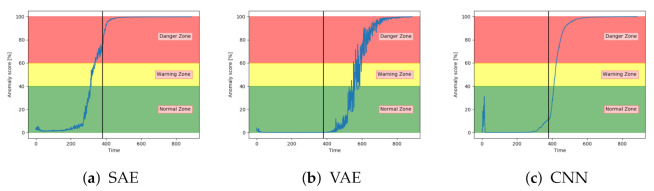
Anomaly score from SAE, VAE, and CNN on sequence from configuration set with failure due to fault type B.

Figure 13a–c show the anomaly score obtained from the SAE, VAE, and CNN models on a sequence from the configuration set with fault type B. The results show that the three models start in the normal zone and increases. The main difference between them was that the VAE and CNN increase towards the maximum score later than the SAE.

All models except DBN show promising results on the sequences from the configuration set. The next step is to evaluate the performance of anomaly detection on unseen data.

## 7. Results

The models were evaluated and tested based on unseen data, which contains four different types of faults. The models were first evaluated based on the accuracy of classifying the 350 test samples into normal or anomalous data. Next, the anomaly score of the complete sequences was visually analysed and interpreted. Table 9 presents the accuracy of each model for all test samples and for each fault type.

The results showed large differences in the classification performance of the models. Both the VAE and LSTM impressively achieved 100% accuracy. CNN performed quite closely, by achieving 96.3% and correctly classifying all samples in the test set with fault type A, C, and D. The three best models were able to perfectly detect all samples within the two new fault types (C and D). The three remaining models (AE, SAE, and DBN) achieved between 78% and 80% accuracy. Table 10 presents an overview of the number of incorrect classifications and how many of them were present in the warning zone.

The AE, SAE, and CNN have most of their miss-classifications in the warning zone, while the DBN has almost every miss-classification in the wrong zone. DBN performed worse than the other models. There were large differences in the classification performance of the models. While VAE, LSTM, and CNN performed with a high accuracy, the other models have several miss-classifications. The anomaly score is not designed or intended to be a pure classifier of normal or failure samples. Therefore the visual analysis is just as important. Next, results from each fault type are analysed.

### Fault A

**Figure 14:**
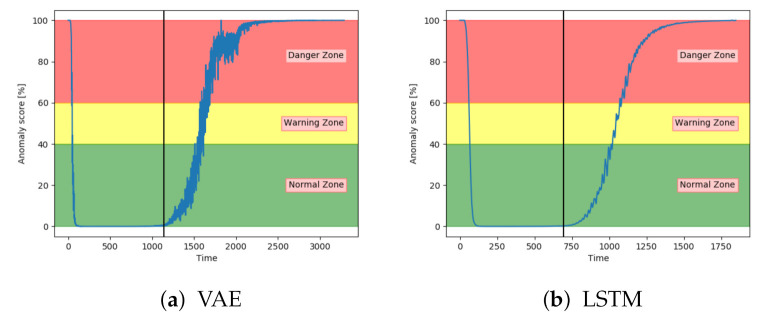
Anomaly scroe from VAE and LTSM on an unseen sequence with fault type A.

Both the VAE and LSTM achieved 100% accuracy on the test set. Figure 14a shows the anomaly score for VAE on one of the unseen sequences with fault type A. Figure 14b shows for another sequence, but with LSTM. The anomaly score started around zero for both models and increased gradually after the 100% fault severity was reached. They were, as desired, able to detect and indicate an anomaly score of 100 in advance of end-of-life. The results were quite similar for all sequences with fault A for these two models.

**Figure 15:**
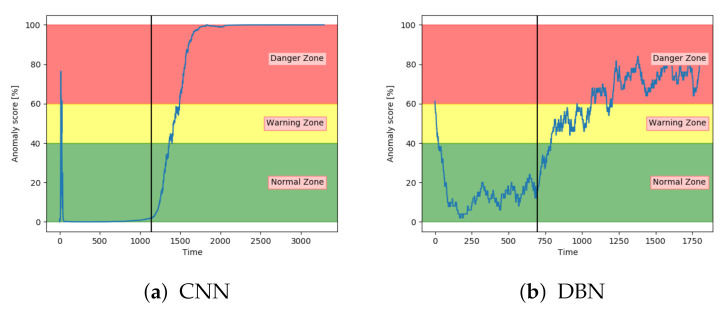
Anomaly score from CNN and DBN on unseen sequence with fault type A.

Figure 15a shows that the CNN performed similarly to VAE and LSTM for fault type A. Figure 15b indicates that DBN was less confident in the normal zone, and increased only slightly into the danger zone. It was enough for the model to get several correct classifications, but it provides worse certainty than the other models. It also has more noise.

**Figure 16:**
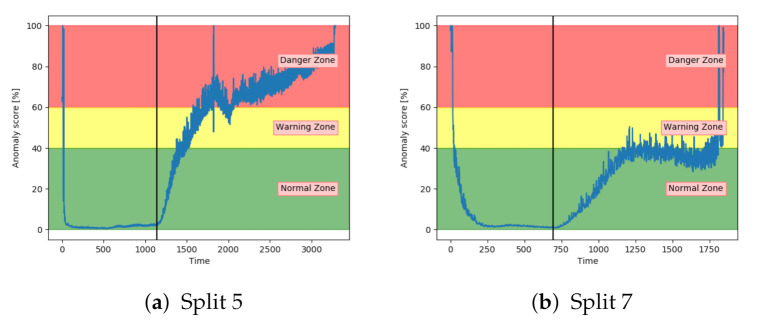
Anomaly score from AE model on unseen sequences with fault type A.

Figure 16a,b shows the anomaly score obtained from the AE for two different sets with fault A. While (a) shows a satisfactory performance, (b) shows that the anomaly score was barely able to leave the normal zone. The AE received inconsistent results for sequences with the same fault type.

**Figure 17:**
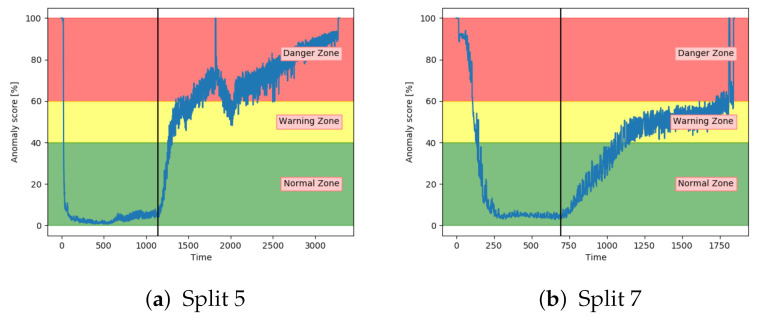
Anomaly score from SAE model on unseen sequence with fault type A.

Similarly to the AE, the SAE achieved inconsistent results. This is indicated in Figure 17. The results on split 7 were better than the AE results, but still not able to properly increase into the danger zone.

Analysis of the anomaly score on sequences with fault type A has indicated that only VAE, LSTM, and CNN achieved the desired results. They were able to indicate anomalous behaviour before reaching end-of-life. The three other models had more fluctuating and inconsistent results.

### 7.1. Fault B

Sequences with fault B was, according to sensor values and domain-experts, affecting the air compressor in a higher degree than fault type A. This means that there was a shorter period from the fault reaching 100% severity, until the system failed.

**Figure 18:**
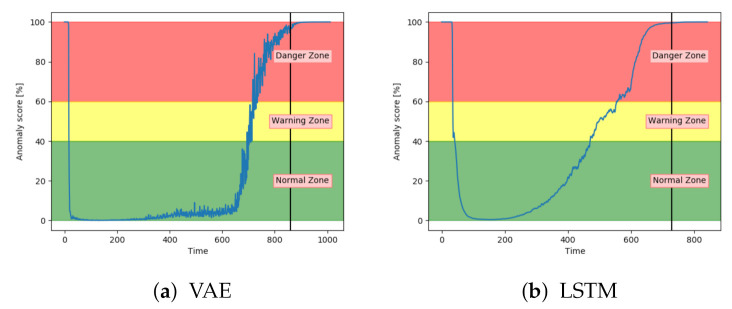
Anomaly score from VAE and LSTM on an unseen sequence with fault type B.

Figure 18a shows that the anomaly score from VAE reaches 100 before the system fails. Compared to results on fault A the anomaly score increased before the 100% fault severity was reached. This supports the information that fault B has larger effects on the system. Figure 18b show similar tendencies from the LSTM model. The main difference was that the anomaly score from the LSTM model started to increase earlier.

**Figure 19:**
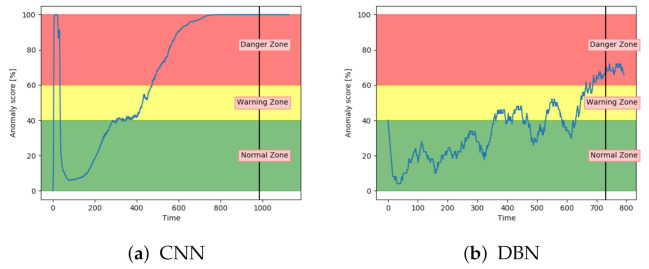
Anomaly score from CNN and DBN on an unseen sequence with fault type B.

The accuracy on the test samples indicated that the CNN model struggled to correctly identify the samples from sequences with fault type B. Figure 19a supports these results by showing that the anomaly score was rarely in the normal zone. The anomaly score increases slowly, but starts too early. According to Figure 19b, the anomaly score from DBN was not able to increase into the danger zone until right before failure. Compared to the other models, the anomaly score from DBN lies much higher in the normal zone and much lower in the danger zone.

**Figure 20:**
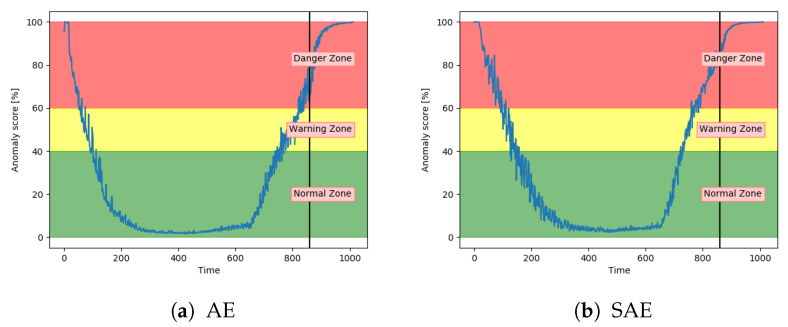
Anomaly score from AE and SAE on an unseen sequence with fault type B.

Figure 20a,b show that both the AE and SAE achieved the desired trend of the anomaly score. For both models, it stayed in the normal zone before gradually increasing up to an anomaly score of 100. Compared to the other models, the results from the AE and SAE started with a longer period in the error zone, before reaching the normal zone. This can either be because of how the sequences are logged or a problem with these models.

Results have indicated that the VAE and LSTM were the best performing models on sequences with both fault type A and B. The CNN showed a promising performance, but was too early on increasing the anomaly score, especially on fault type B. The DBN was not able to properly increase the anomaly score into the danger zone and had many fluctuations. Both the AE and SAE provided better results for fault B, than A.

### 7.2. Fault C

**Figure 21:**
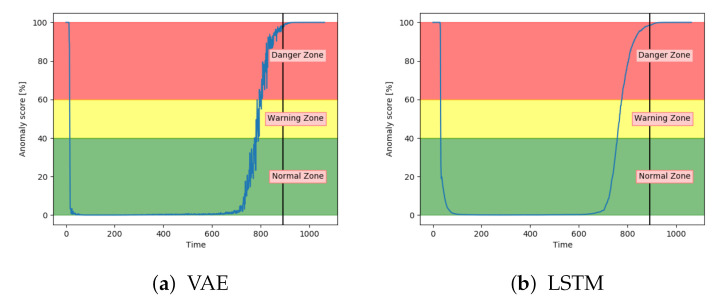
Anomaly score from VAE and LSTM on an unseen sequence with fault type C.

The performance of the models were also analysed on a new type of fault, not present in either training or configuration. This can show if the algorithm can generalise and detect anomalous behaviour caused by new, unseen types of faults. The two best performing models so far are the VAE and LSTM. Figure 21a,b show that both of these models achieved the desired behaviour and could give a clear indication of anomalous behaviour before the system fails.

**Figure 22:**
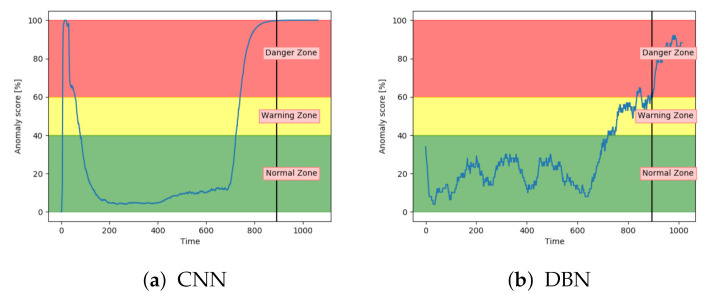
Anomaly score from CNN and DBN on an unseen sequence with fault type C.

Figure 22a shows that the CNN model was able to detect anomalous behaviour on a sequence with fault type C before the system fails. It performs similarly to the VAE and LSTM. Compared to previous results, the DBN were able to detect anomalous behaviour on the sequence with fault type C. It has an anomaly score which fluctuates more than for other models, but it stays within the appropriate zones and gives an indication of anomalous behaviour before the system fails.

**Figure 23:**
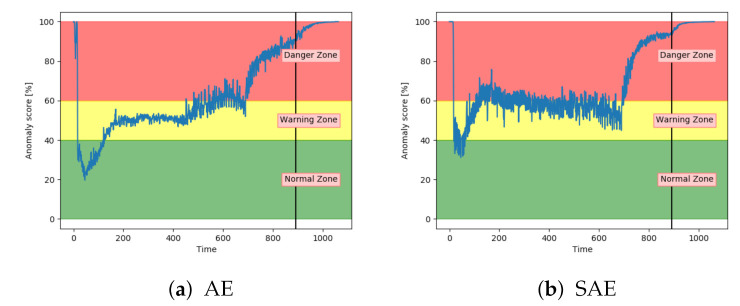
Anomaly score from AE and SAE on an unseen sequence with fault type C.

Figure 23 indicates that the AE and SAE were not able to generalise. The anomaly score stayed in the warning zone, instead of the normal zone. They were both successful in detecting an anomaly score at 100 before system failure. They were, on the other hand, unsuccessful in capturing that the system was in the normal zone for most of the sequence.

### 7.3. Fault D

**Figure 24:**
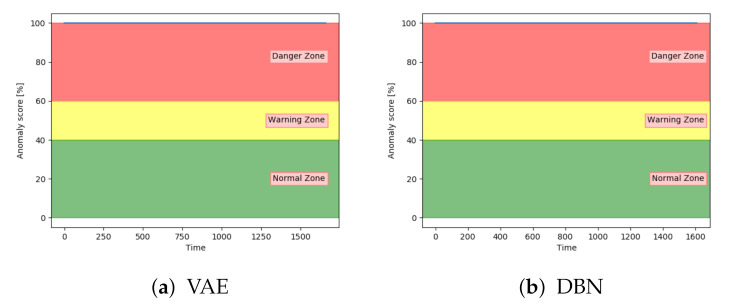
Anomaly score on an unseen sequence with fault type D.

The models were analysed based on the performance on sequences with fault type D. Compared to the other fault types, this fault type has the same severity through the entire sequence. The results from the classification of the test samples showed that every model was able to accurately classify between normal and anomalous samples on sequences with fault type D. Visual analysis showed that all models keep an anomaly score of about 100 through the entire sequences. Figure 24 proves this by showing the anomaly score on a sequence with fault D with both VAE and DBN. The other models follow the same pattern.

### 7.4. Model Evaluation

The results have proven that VAE and LSTM can accurately detect anomalous behaviour in air compressors. Both models showed promising results in classification and visual analysis. CNN did not perform as well as VAE and LSTM. It received a high accuracy on the test samples, and visual inspection proved that the CNN is promising. The three remaining models achieved lower accuracy on the test samples and were only able to deliver suitable anomaly scores on some of the fault types and sequences. The results and model evaluation are discussed more later.

Anomaly detection is typically a black box, only indicating if data are anomalous or normal. The proposed method based on anomaly score can indicate how much the behaviour is deviating from normal condition. This increases the transparency compared to typical anomaly detection approaches. In the next section, a method to make the anomaly score more transparent is proposed.

## 8. Model Transparency

A low anomaly score shows that the system is acting close to normal behaviour. An increasing anomaly score indicates that something unexpected is happening. When the anomaly score goes towards 100, it can be interpreted as a danger for failure. It indicates that the system is behaving anomalously and might fail. The anomaly score gives no information about what is wrong with the system, only that something is wrong. Historically, anomaly detection is not used for this since its purpose is mainly to discover anomalies, while diagnostics will uncover the actual fault. Diagnostics is dependent on having examples of previous faults. The proposed method for increasing the transparency in the anomaly score can give an alternative, which can help to identify faults without historical examples.

The proposed method for increasing the transparency is to calculate each input’s (in this case, each sensor’s) contribution to the anomaly score. Since all inputs are normalised between the same range, they can influence the score equally. The mean absolute error (MAE) is the mean of the absolute error between each pair of input and reconstructed input. Therefore, by not taking the mean of the errors, the error of each input in the sample is known. The contribution can then be found by dividing the error of one sensor, by the total error. Equation (3) shows how the contribution of one input, from one sample, can be calculated. In the equation, *i* indicates the sample and *j* indicates the input (sensor):

(3) $$\mathrm{Contribution}\;{\mathrm{Input}}_{i,j}=100*\frac{|Input_{i,j}-\hat{Input_{i,j}}|}{\mathrm{Reconstruction}{\mathrm{Error}}_{i}}$$

The proposed method adds information to the anomaly score that indicates which sensors in the signal contribute the most to the reconstruction error. The idea is that if a domain expert or service personnel see the top deviating sensors, the potential faults can be recognised. The method works for all the proposed models. Next, some examples of the anomaly score and the belonging contribution are shown. Due to the confidentiality requirements, the sensors are named with numbers (S1, S2, etc.). In reality, the sensor names have a description telling what is being measured (temperature, pressure, etc.) and where it is measured.

**Figure 25:**
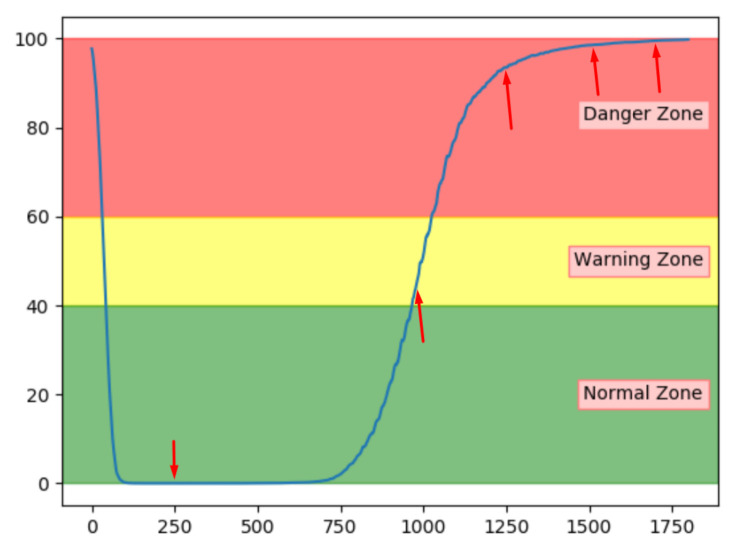
Selected test samples from sequence with fault A.

First, five samples from a sequence with fault type A were explored. The chosen samples range from normal to failure condition. They are marked in Figure 25. Table 11 shows the top three sensor contributions for each of the samples. The first sample, which is considered to be in normal operation condition indicates that sensor S3 is the main contribution to the anomaly score. The four remaining samples show an increasing contribution from sensor S6. This makes the anomaly score more transparent and lets a domain expert have an idea of potential faults that are occurring. The exact position of the sensor related to S6 can tell if it is related to an engine, cooling, or other faults. The contribution of each sensor does not make sense for a low anomaly score. It should only be used together with an anomaly score outside of the normal zone, to investigate the source of the issue in the system.

**Figure 26:**
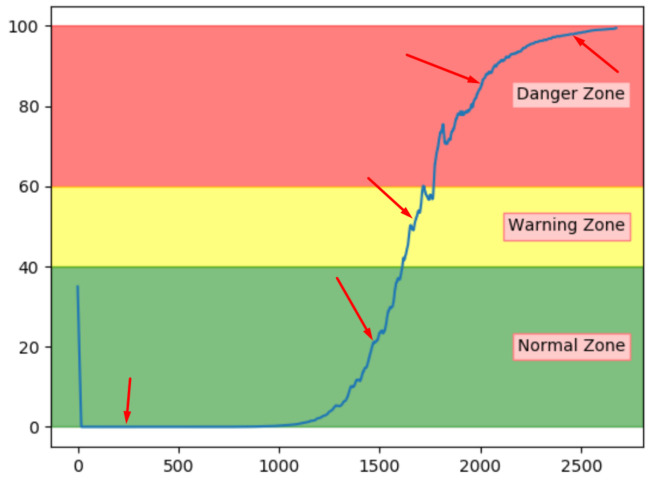
Selected test samples from another sequence with fault A.

Another example of a sequence with fault A was explored to see if the sensor contributions are helpful. This should provide results that indicate a similar trend as the previous example. Figure 26 shows the selected samples. According to Table 12, the contributing sensors are following a similar trend for this sequence, where S6 has an increasing contribution. The contributions from the normal sample can be ignored since the anomaly score is quite low and therefore has no fault to investigate.

**Figure 27:**
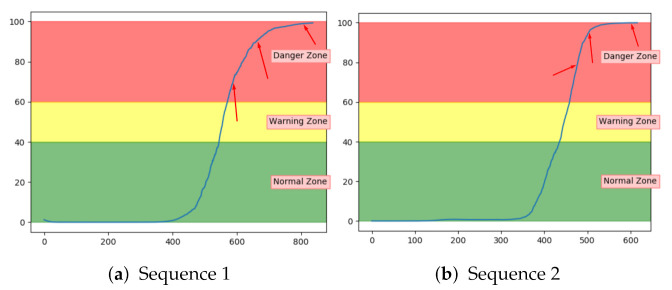
Selected samples for sensor contribution from sequences with fault B.

Thus far, the proposed method to increase transparency seems promising. The next step is to see if a sequence with another fault type has a unique pattern of top contributing sensors. Figure 27 shows two sequences with fault type B, and the red arrows indicate the three samples that were investigated. Since the normal samples can be considered as insignificant, only error samples are included.

Table 13 shows that the samples from the sequence in Figure 27a indicate sensor S3 as the main contributor to the error. Similarly, Table 14 shows that S3 was also the main contributor to the other sequence. In addition, the tables show that for both sequences, S5 was also contributing to the anomaly score. This can give service personnel an indication that the fault is somehow related to these two sensors.

The proposed method has proved useful in increasing the transparency in the anomaly detection approach. It uncovered that each fault type has a unique pattern of top contributing sensors. These can be used to indicate why the system is behaving abnormally.

## 9. Discussion

The results show that it is possible to detect anomalous behaviour in air compressors and use it to indicate system health. All six DL techniques (AE, SAE, VAE, DBN, ED-LSTM, and ED-CNN) were able to reconstruct normal data with a relatively low error. The reconstruction error increased when faults were introduced and as they progressed. When the models were tested on sequences with faults, the reconstruction error started at a similar level as normal sequences but increased as desired. The main difference between the models was the level of noise in the reconstruction error. Results from DBN had a lot of noise, while LSTM and CNN achieved results with little noise.

One of the main difficulties with the reconstruction-based approach was that it is challenging to tune. If a model achieves perfect reconstruction of normal data, it does not give insight into data performance with faults. If a hyper-parameter optimisation loop is used, which is uncritical for such a problem, a risk is that the obtained architecture allows the input to be copied to the output. This will achieve almost perfect reconstruction on both normal and fault data, not the desired behaviour. The architectures and hyper-parameters were therefore explored through trial and error. The manual process tested several architectures until the desired behaviour was achieved. If the process is to be automated, a good evaluation method must be found.

Anomaly detection was explored towards giving a descriptive range of how much an air compressor deviates from the normal operating condition. The proposed method transformed the raw reconstructions into a more descriptive range, referred to as the anomaly score. The reconstruction error was in different ranges for each of the DL models. It was therefore anticipated to be difficult to find a common and descriptive scale of the reconstruction error. The proposed transformation contained three steps (scale, sigmoid transformation, and scale), which were tuned based on a configuration set. It proved to give a suitable scale, giving an anomaly score that ranged from 0 (normal condition) to 100 (close to failure). The drawback with the approach is that it requires manual tuning and is sensitive to changes. If little data is available to configure the transformation, an alternative is to use the reconstruction errors directly. The raw reconstruction error and its historical values can help to indicate anomalous behaviour. If it is increasing, it can indicate that there is something wrong with the system. The anomaly score’s advantage is that a single value is much more descriptive than the raw reconstruction error. Both of these methods would work together with the proposed transparency method.

The range [0,100] was divided into three zones (normal, warning, and danger) to get more information from the anomaly score. These zones were tuned manually to classify normal data in the normal zone and data with faults in the danger zone. While manually tuning such thresholds is a drawback, the advantage is the flexibility of tuning it to the desired behaviour. A potential user can decide if it is desirable to get early warnings or wait until the anomalous behaviour is more guaranteed. In this research, the zones and threshold are used to provide a clearer insight into how the proposed approach can be used, but they can be replaced if the reconstruction error with the change rate is used directly. The proposed anomaly score approach is not intended to be used purely as a classifier. The thresholds can, on the other hand, be useful for giving warnings and error.

One of the problems with the proposed anomaly score approach is that it is hard to evaluate. The models were therefore evaluated in two ways: Visually and based on classifications from the thresholds. The classification accuracy is not an adequate method to evaluate the models and the visual inspection is not quantitative and therefore, hard to automate. The combined evaluation is sufficient and possible for this case, and small datasets, but better evaluation methods should be explored. All evaluation was done on unseen data with four different fault types. Two of the fault types were not represented in either training or configuration. Visual inspection indicated that the anomaly score obtained from LSTM, CNN, and VAE was the most promising. They were able to give a clear indication that the air compressor deviated from the normal operational condition in a reasonable time before failure. The three remaining models performed worse and struggled to give clear and consistent results. The classification was done on a set of partly randomly selected samples and supported that LSTM, CNN, and VAE performed the best. Both VAE and LSTM achieved 100% accuracy. Interestingly, two out of the three best performing models use time windows, while the three worst works with non-sequential data. The VAE differs from the other models by modelling probability. The results indicate that VAE and ED-architecture LSTM are great choices for anomaly detection on air compressors.

The proposed method for making the anomaly score more transparent indicates why an air compressor deviates from the expected behaviour. A unique pattern of top contributing sensors for each fault type was detected. Service personnel can, based on the top contributing sensors, identify the potential fault or at least which parts of the system are causing the deviation. Increased transparency in anomaly detection makes such a feature much more useful. The contributing sensors have little significance when the anomaly score is in the normal zone. The contributing sensors should only be investigated in combination with an anomaly score outside of the normal zone. It is essential to evaluate the scalability of the proposed method. The air compressor explored in the thesis has few sensors, but the method is developed to scale to larger systems with several sub-systems. In such cases, it is possible to build a tree-structure describing the relation between sensors. Each sub-system can have its branch. In such cases, the method can be tuned to indicate the top contributing sub-system and the contributing sensors within them.

It is important to consider if the proposed method for anomaly score with transparency is useful. It has already proven to detect that the air compressor deviates from the normal condition and indicates why it deviates. The greatest advantage of the approach is that it is only trained on data in the normal operating condition. It is beneficial to have a few run-to-failure examples of tuning the model. If these are not available, the reconstruction error can be used directly until the required data are collected. This means that a company can easily start with this feature in a PHM system without having large quantities of labelled data. As mentioned in the introduction, a key challenge with industrial data, particularly data from critical equipment, is the lack of examples of anomalous behaviour. Moreover, if such examples exist, they are often not labelled. This is what anomaly detection is used for however, it is challenging to achieve provably significant results. More data must be gathered and analysed further to verify the significance and value of the proposed solution. This early version can be put in use to enhance data collection to achieve that. The predictions can be made available only to trained service personnel to put it in use safely, who can inspect and verify the results before notifying the customer. The results can be verified before being presented to the end-user and can aid in labelling the data when fault and abnormal behaviour occur.

In addition, the approach proved to detect anomalous behaviour for unseen types of faults. This indicates that compared to fault diagnostics, this approach can detect something wrong in the system without having historical examples of that specified fault. A common problem in the industry, especially the maritime industry, is few run-to-failure examples and little labelled data in general. This approach can still be used in such cases. When more data are collected, the approach can be re-configured, improved, and evaluated. The anomaly score approach can answer the question: “Is there something wrong with the air compressor?”.

## 10. Conclusions

Detecting abnormal behaviour in the air compressor system was done by exploring six different DL methods based on an ED principle. The models were used to try to reconstruct raw input data through a lower-dimensional latent space. The proposed method transformed the reconstruction error from the models into a descriptive range referred to as the anomaly score. Visual inspection showed that the anomaly score from VAE and LSTM was performing best. They were able to give a strong indication of how much the air compressor deviated from normal operational conditions. Both models were able to indicate highly anomalous behaviour in advance of failures. The same two models proved to accurately identify if a compressor was in a normal or faulty operating condition. A method based on error contribution was proposed to increase the transparency of the anomaly score. It led to the anomaly score indicating the condition of the system and which parts of the system were contributing to the deviation. The proposed approach could help avoid that the equipment fails silently and notify the equipment that the equipment is behaving unexpectedly. The transparency extension could even help to indicate which part of the system was being affected. The proposed methods can be highly valuable as a starting point to PHM, as it requires less labelled data than typical data-driven approaches to diagnostics and prognostics.

### Future Work

The proposed approach showed promising results on a small dataset with normal and fault data from maritime air compressors. It is suggested that future work should apply the approach on other, and more complex equipment to contribute to verifying the usefulness of the approach.

One of the mentioned drawbacks of the proposed method is the manual tuning and selection done in this research. As described in the methodology, several of these manual processes, such as zone-selection and range transformation, are avoidable. Instead of using hard thresholds in a standardised range, the rate of change in the reconstruction error can be monitored directly.

Furthermore, we propose that future work should look at how to automate and pipeline the entire process, from training to deployment. One of the key challenges is to automatically train and tune the model architecture without ending up with a model that allows the input to be copied to the output, as described in the discussion. Therefore future work needs to explore and find a suitable scoring function that sufficiently measures the performance of approaches that tries to indicate unexpected behaviour and potential health of equipment.

## Figures and Tables

**Table 1:** Description of available data and its usage.

Type	# Datasets	Sequence Length
Normal	8	77–1250
Fault A	7	909–3643
Fault B	7	666–2554
Fault C	1	1064–1064
Fault D	2	1228–1660

**Table 2:** Selected parameters for AE.

Auto-Encoder-PARAMETERS	
Optimiser	RMSProp
Learning rate	0.0001
Input Layer	14
H1-H5 Layer	17-8-4-8-17
Output Layer	14

**Table 3:** Selected parameters for sparse AE (SAE).

Sparse Autoencoder-Parameters	
Optimiser	RMSProp
Learning rate	0.0001
Input Layer	14
H1-H5 Layer	14
Output Layer	14

**Table 4:** Selected parameters for variational autoencoder (VAE).

Variational Autoencoder-Parameters	
Optimiser	Mini-batch SGD
Learning rate	0.001
Input Layer	14
H1-H5 Layer	16,8,6,8,16
Output Layer	14

**Table 5:** Selected parameters for DBN.

Deep Belief Network-Parameters	
Learning rate	0.003
*k*	10
Batch size	25
Input Layer	14
Hidden Layers	16,13,11

**Table 6:** Selected parameters for long short-term memory (LSTM).

Long Short-Term Memory-Parameters	
Optimiser	SGD
Learning rate	0.001
Batch size	100
Time window	20
Input Layer	14
Hidden Layers	10, 7, 10
Output Layer	14

**Table 7:** Selected CNN-architecture and parameters.

Convolutional Neural Network-Parameters	
L1: Conv2D	16 filters and 8x8 kernel. ReLU activation function.
L2: Maxpooling	Pool size (2,2)
L3: Conv2D	8 filters and 3x3 kernel. ReLU activation function.
L4: Maxpooling	Pool size (2,2)
L5: Conv2D	8 filters and 3x3 kernel. ReLU activation function.
L6: Upsampling2D	Size (2,1)
L7: Conv2D	16 filters and 3x3 kernel. ReLU activation function.
L8: Upsampling2D	Size (2,2)
L9: Conv2D	1 filter and 8x8 kernel. Tanh activation function.

**Table 8:** Anomaly score transformation parameters for each model.

	Old Min	Old Max	New Min	New Max	Sigmoid Exponent
AE	0.01	0.18	−8	9	0.8
SAE	0.01	0.16	−8	9	0.6
VAE	0.205	0.32	−8	9	0.8
DBN	0.452	0.458	−8	9.5	0.7
LSTM	0.15	0.27	−9	9	0.4
CNN	0.05	0.20	−8	8	0.4

**Table 9:** Total accuracy and accuracy per fault type for each deep learning (DL) model.

	Accuracy				
**Model**	**Total**	**Fault A**	**Fault B**	**Fault C**	**Fault D**
AE	0.797	0.8	0.99	0.5	1.0
SAE	0.783	0.81	0.93	0.5	1.0
VAE	1.0	1.0	1.0	1.0	1.0
DBN	0.794	0.83	0.65	0.8	1.0
LSTM	1.0	1.0	1.0	1.0	1.0
CNN	0.963	1.0	0.87	1.0	1.0

**Table 10:** Miss-classifications in the anomaly detection models.

Model	# Misses	# In Warning Zone	Share in Void
AE	71	60	0.85
SAE	76	58	0.76
DBN	72	2	0.03
CNN	13	11	0.85

**Table 11:** Top sensors contributing to the anomaly score on samples from Figure 25.

	Sample #1	Sample #2	Sample #3	Sample #4	Sample #5
Top 1 Contributor	S3: 38.85%	S6: 25.09%	S6: 29.29%	S6: 30.63%	S6: 32.13%
Top 2 Contributor	S1: 20.38%	S5: 19.30%	S5: 17.78%	S1: 17.83%	S1: 19.41%
Top 3 Contributor	S8: 10.93%	S1: 14.66%	S1: 16.11%	S5: 17.52%	S5: 17.30%

**Table 12:** Top sensors contributing to the anomaly score on samples from Figure 26.

	Sample #1	Sample #2	Sample #3	Sample #4	Sample #5
Top 1 Contributor	S3: 27.85%	S6: 32.14%	S6: 32.92%	S6: 33.33%	S6: 33.71%
Top 2 Contributor	S1: 18.44%	S5: 18.75%	S5: 19.64%	S5: 19.14%	S5: 19.38%
Top 3 Contributor	S6: 16.87%	S1: 10.45%	S1: 12.70%	S1: 14.50%	S1: 17.73%

**Table 13:** Top sensors contributing to the anomaly score on samples from Figure 27a.

	Sample #1	Sample #2	Sample #3
Top 1 Contributor	S3: 30.4%	S3: 28.50%	S3: 29.38%
Top 2 Contributor	S5: 14.58%	S5: 15.28%	S5: 16.11%
Top 3 Contributor	S1: 11.22%	S4: 11.83%	S4: 12.19%

**Table 14:** Top sensors contributing to the anomaly score on samples from Figure 27b.

	Sample #1	Sample #2	Sample #3
Top 1 Contributor	S3: 17.23%	S3: 20.41%	S3: 24.99%
Top 2 Contributor	S5: 17.10%	S5: 16.52%	S5: 14.56%
Top 3 Contributor	S1: 16.97%	S1: 14.51%	S1: 13.29%

## Data Availability

Data sharing not applicable.
